# Labor and delivery service use: indigenous women’s preference and the health sector response in the Chiapas Highlands of Mexico

**DOI:** 10.1186/s12939-015-0289-1

**Published:** 2015-12-23

**Authors:** Midiam Ibáñez-Cuevas, Ileana B. Heredia-Pi, Sergio Meneses-Navarro, Blanca Pelcastre-Villafuerte, Miguel A. González-Block

**Affiliations:** Centre for Health Systems Research, National Institute of Public Health, Avenida Universidad # 655, Colonia: Santa María de Ahuacatitlán, CP: 62100 Cuernavaca, Morelos Mexico; Regional Centre for Public Health Research, National Institute of Public Health, Tapachula, Mexico

**Keywords:** Indigenous population, Service utilization, Midwives, Intercultural birth, Vaginal examinations

## Abstract

**Background:**

Mexico has undertaken important efforts to decrease maternal mortality. Health authorities have introduced intercultural innovations to address barriersfaced by indigenous women accessing professional maternal and delivery services. This study examines, from the perspective of indigenous women, the barriers andfacilitators of labor and delivery care services in a context of intercultural and allopathic innovations.

**Methods:**

This is an exploratory study using a qualitative approach of discourse analysis with grounded theory techniques. Twenty-five semi-structured interviews were undertaken with users and non-users of the labor and delivery services, as well as with traditional birth attendants (TBAs) in San Andrés Larráinzar, Chiapas in 2012.

**Results:**

The interviewees identified barriers in the availability of medical personnel and restrictive hours for health services. Additionally, they referred to barriers to access (economic, geographic, linguistic and cultural) to health services, as well as invasive and offensive hospital practices enacted by health system personnel, which limited the quality of care they can provide. Traditional birth attendants participating in intercultural settings expressed the lack of autonomy and exclusion they experience by hospital personnel, as a result of not being considered part of the care team. As facilitators, users point to the importance of having their traditional birth attendants and families present during childbirth, to allow them to use their clothing during the attention, that the staff of health care is of the female sex and speaking the language of the community. As limiting condition users referred the different medical maneuvers practiced in the attention of the delivery (vaginal examination, episiotomy, administration of oxytocin, etc.).

**Conclusions:**

Evidence from the study suggests the presence of important barriers to the utilization of institutional labor and delivery services in indigenous communities, in spite of the intercultural strategies implemented. It is important to consider strengthening intercultural models of care, to sensitize personnel towards cultural needs, beliefs, practices and preferences of indigenous women, with a focus on human rights, gender equity and quality of care.

## Background

Maternal mortality continues to be an important public health issue that illustrates profound social inequities in poor and middle income countries [1]. Mexico has undertaken important efforts in recent years to decrease maternal mortality [2, 3]. However, Mexico did not reach the Millenium Development Goal of reducing the maternal mortality ratio by three-quarters. The current maternal mortality ratio is 38.3 per 100,000 live births and the country faces major challenges in order to achieve strong results related to the sustainable development goals (SDG) in this indicator [4, 5]. The problem continues to be one of the primary expressions of health inequity, particularly among highly vulnerable groups such as the indigenous population [3]. Among indigenous women, four levels of vulnerability are manifested: poverty, ethnic discrimination, gender discrimination and geographic inaccessibility to health services [6].

Chiapas is among the states of Mexico with highest concentration of indigenous population (27.2 % of the total) and with 32.5 % not speaking Spanish [7]. In 2012, Chiapas reported a maternal mortality ratio (MMR) of 59.8 per 100,000 live births, 40 % above the average and the third highest in the country [4].

To change these indicators, health policies have been designed to increase access to and utilization of health services, particularly professional care related to labor and delivery, and obstetric emergencies [2]. These efforts have been specifically focused on the poorest municipalities of the country with an emphasis on indigenous regions [2]. As a result, the percentage of labor and delivery care provided by qualified personnel in Chiapas has increased from 42 % in 2007–55 % in 2011 [8]. However, this increase has failed to meet the goal of 92 % of professional delivery care established by national authorities in the 2007–2012 health sector programs [8].

Health authorities in Chiapas have introduced intercultural innovations to address barriers faced by indigenous women accessing professional maternal and delivery services [9]. More specifically, providers’ capacity has been strengthened to offer vertical birthing as an alternative to the horizontal position, as well as to incorporate symbolic elements and traditional interventions such as crosses or *temascal* the traditional steam bath with medicinal herbs [9]. The inclusion of trained traditional birth attendants (TBAs) into the institutional prenatal and delivery care process has been strengthened, giving them also a role to promote health service access [9]. One such initiative has been the construction of *Casas Maternas* (Maternity Homes in English, hereafter referred to as CM) adjacent to health centers that offer expanded services or basic hospitals centers [10]. The objective of the CM is for women to be able to receive labor and delivery care in accordance with their preference, in the birthing position they choose, attended to by TBAs, with the support of their family and ritual or symbolic elements from their own culture in settings with adequate hygienic conditions and timely access to effective medical assistance in the event of any complications [10]. One of these CM was established in 2010 [11] in the municipality of San Andrés Larráinzar, adjacent to the local basic hospital.

Evidence suggest that in spite of intercultural innovations, a significant number of women and their family members (husband, mother-in-law, mother, father) decide to not seek care at the health center for labor and delivery, and instead decide to receive care in their homes by TBAs or family members. Among the primary reasons for this preference is the husband’s negative perception of his wife being observed and probed by male health workers. This barrier to care stems from stereotypes of western doctors and hospital, the perception of discriminatory attitudes and treatment, shame related to nudity and genitals touched by strangers, the distance between their communities and the health centers, and economic limitations [12–14].

Intercultural innovations can thus play a critical role to facilitate access to professional maternal and delivery services. Studies on the determinants of successful implementation of innovations in health has highlighted the understanding of innovation characteristics, the receptivity and support offered by the internal and external contexts where innovations are implemented, as well as the definition and understanding of roles played by actors and processes during implementation [15]. This article describes the perceptions of indigenous users and non-users and of traditional birth attendants concerning facilitating and obstructing factors in the implementation of intercultural maternal health innovations. Research focused on the characteristics of the innovations and the internal context from the perspective of users as key actors in implementation. The innovations were analyzed from the perspective of the perceived quality and acceptability of medical services offered at the CM and the basic community hospital. The internal context was addressed by analyzing perceptions on the conditions related to availability and accessibility, and on the determinants of utilization.

## Methods

### Study design

A qualitative study was designed to explore in-depth the factors that play a role in indigenous women’s decision about what type of care they prefer during delivery and their perceptions about institutional health services.

### Participants’ selection

The selection of the participants was by previously defined criteria [16]. This strategy allowed us to define the profile of the participants according to the study objectives. The total number of participants was determined by theoretical saturation [17].

### Data collection

In total, semi-structured interviews with 25 indigenous women with deliveries in the past year were conducted: six who had contact with the CM during their last pregnancy or delivery in the past year; seven who had delivered in the last year but did not have any contact with professional health service providers, and twelve local TBAs affiliated with the CM. An interview guide designed specifically for this study was used to obtain information about the perceptions of indigenous women and TBAs in regards to labor and delivery care at the CM and the hospital as well as the availability and conditions of access. The total number of interviews was sufficient to achieve informational richness according to the aims of the study [18].

### Study setting

The data used for this analysis were collected in 2012 in the CM adjacent to the Basic Community Hospital in San Andrés Larráinzar in the Chiapas highlands. This hospital has 12 beds for hospitalization, general surgery services, obstetrics and gynecology, pediatrics, a clinical laboratory and pharmacy, among other services. The CM*,* adjoined to the hospital by a hallway that leads to the operating room, has two large rooms with individual beds used for patient care, two bathrooms, one kitchen with a wood-burning stove and another with a gas stove, a waiting room and a garden with a *temascal* and medicinal plant garden. The CM is run by an indigenous nurse but all services provided to women are delivered by a TBA in cooperation with the institutional health services; in the event of a complication the TBAs request support from the hospital’s medical personnel.

### Ethical considerations

This study was approved by the research and ethics committees of the National Institute of Public Health. The interviews were conducted after verbal informed consent was given by informants. The majority of interviews were conducted in the *Tsotsil* language with the support of a bilingual indigenous interpreter. The interviews were audio-recorded, and subsequently translated to Spanish by the interpreter, transcribed and entered into an Excel matrix designed specifically for the analysis of data collected as part of this study.

### Data analysis

The method used for analyzing the data collected in this study was based on discourse analysis with procedures of grounded theory. Grounded theory was used to conduct the analysis only through the processes of categorization, coding, analysis and interpretation of data without seeking the construction of a theory [19–21].

Discourse analysis encompasses the set of methods and procedures used in data analysis which help identify the meaning of phrases, testimonials or texts within context [19–21]. The following analytic categories were defined to organize the data: availability, accessibility, acceptability and quality of maternal health services. For this study the categories were conceptualized as follows:

#### Availability

Perceptions related to the number of establishments, goods, services and labor and delivery personnel [22, 23].

#### Accessibility

This includes the interviewee’s perception of access to health services along four dimensions: a) geographic, b) financial, c) organizational and d) cultural [22, 23].

#### Acceptability

Perceived respect and adaptability of the health services organization in regards to the population’s needs and priorities [22, 23].

#### Quality

This includes interviewed women’s expressed opinions about medical personnel, the establishment, good and services, as well as perceptions of the care interviewees received at these institutions [22, 23].

## Results

We present the results organized by thematic categories with illustrative participant quotes, as follows,

### Socio-demographic profiles of indigenous women users, non-users and traditional birth attendants

Six users of health services were included in the study. By user of health services we mean women who, during pregnancy, birth or post-partum, had contact with institutional health services, not necessarily that they were seen by institutional personnel throughout the entire process. The mean age for this group was 26 years and had on average 3 children. Similarly, seven non-users of health services were interviewed and 12 community-based TBAs affiliated with the health services were interviewed. Table 1 shows socio-demographic profiles of indigenous women users, non-users and traditional birth attendants.

**Table 1:** Socio-demographic profiles of indigenous women users, non-users and traditional birth attendants

	*Type of actor*		
	Users (*N* = 6)	No users (*N* = 7)	TBAs (*N* = 12)
*Sociodemographic characteristics*			
Age (years: average)	26.1	25.4	61.3
Children (per woman)	3.1	3.5	N/A
*Language*			
Spanish*	1/6	0/7	0/12
Tsotsil	5/6	7/7	12/12
Little spanish	5/6	5/7	4/12
*Experience practicing midwifery*			
Years (average)	N/A	N/A	38.8

*Mastery of Spanish

### Perceptions about the institutional delivery care process

#### Perceptions about the availability of health services

The interviewees identified limitations to the availability of medical personnel and the restricted hours of operation at the health services. Users expressed that they did not seek health services for labor and delivery because they did not trust they would find the hospital personnel, due to their work schedules being limited or not being at the worksite or not present at every shift.

*“The doctors only see us when they are on shift, because if you arrive and they are leaving, sometimes they won’t see you, the majority of them say that the next shift is starting” (User, 23 years, 4 children)*

Non-users perceived limited capacity to supply labor and delivery services. They referred to their perception of, for example, if all pregnant women sought care at the hospital for delivery, the hospital would not have the capacity to see all the women.

*“If we all go to the hospital it would be very full, and the doctors would not be able to see us” (Non-user, 30 years, 4 children)*

#### Perceptions about the accessibility of health services

Among the interviewees several coincidences were identified related to the limitations to accessible institutional labor and delivery services. Among the primary factors were linguistic barriers. Beyond the evident problem of lack of basic communication, this results in feelings of fear, distrust, anxiety about the mutual lack of understanding, and vulnerability on behalf of the indigenous women who seek health services.

*“Everything is in Spanish, I understand a little bit [of Spanish], but I would like it if they spoke to us in Tsotsil because we don’t understand [Spanish] very well. I would like it if everything was in Tsotsil so that we could understand what they are trying to explain, in Spanish there are words I don’t understand, even though I tell them it’s ok, but I don’t really know if they understand me either. That’s the reason that most of us don’t ask questions, that’s why we are scared to go to the doctor” (Non-user, 22 years, 3 children)*

Another important barrier to access services is the costs associated with seeking care, particularly related to transportation and food costs for family members that accompany the patient and have to wait during her hospital stay.

*“Additionally, more money is spent when we go to the hospital because we have to pay for transportation, food and we have to have food for our family that accompanies us to the hospital, so it’s a big expense” (Non-user, 22 years, 3 children)*

Service users mentioned that the distance they had to travel from their homes to the hospital was a limitation.

*“We have to start by walking about 20 minutes to be able to find a car by the bridge and then travel another 20 minutes more to the Larráinzar hospital” (User, 30 years, 4 children)*

#### Perceptions about acceptability and quality of health services

A highly reiterated reason to avoid seeking obstetric services was the perceived intimate and invasive nature of vaginal examinations by doctors, particularly male doctors.

*“The doctors are constantly examining you. I don’t know what it’s called when they put their hand in your vagina that is what I do not like. I was very embarrassed, because I am not used to having my body looked at by doctors but what can we do about it once we are in the hospital?(…)” (User, 36 years, 5 children)*

Users occasionally expressed that the health service personnel had used phrases and expressions when referring to them that they had perceived as undignified care, demonstrated little sensitivity and no empathy in regards to the situation the patients were experiencing. This was detrimental to the perceived quality of care and may be considered as an example of disrespect and abuse in childbirth [24, 25].

*“They told me not to push, just lie there, while they were walking around, not supporting me. I felt very bad; I didn’t have the strength because I was there alone. I felt very bad, at that moment I wanted to leave the hospital (…)” (User, 25 years, 2 children)*

Non-users expressed that one of the reasons why they did not seek labor and delivery services at the hospital is because they are required to undress and put on a robe, without explaining why or asking them if they agree with this procedure or not.

*“The take our clothes off, that’s what the doctors do at the hospital, (…) they don’t even ask if you want to put on the type of clothes they wear, they toss our clothes to the side, sometimes we can’t even find our clothes, they get lost (Non-user, 22 year, 3 children)*

The women interviewed considered that only in the event of a complication during pregnancy or labor should they utilize the health services.

*“While I am [giving birth] well, why would I go to the hospital? Only if it’s a serious situation then maybe my family would bring me to the hospital but only because I am doing very badly” (Non-user, 22 year, 3 children)*

Non-users expressed their disagreement with some of the procedures that doctors conduct. According to their testimonials, after assisting with births at the hospital, the doctors sometimes place without informed consent an intrauterine device (IUD), so that women will not have more children. For similar reasons, women fear seeking services at the hospital because of the rumors they have heard about the practice of conducting tubal ligation without informed consent.

*“There [in the hospital] sometimes they don’t ask if you want or don’t want family planning, sometimes they put a device [IUD] in, they don’t even ask. That’s what they say happens, that’s why I don’t like to go to the hospital” (Non-user, 19 year, 1 child)*

### Perceptions about alternative labor and delivery services

#### Care provided by TBAs in community settings

In contrast with the perceptions that interviewees had regarding institutional health services, they identified more strongly with the services offered by community-based TBAs, and preferred them over medical personnel. The reasons they preferred care provided by TBAs was because they spoke the same language, shared their values and world view regarding reproduction, and they did not have expenses associated with transportation to the site of care, since the TBA transports herself to the woman’s home. Furthermore, at the woman’s home, in contrast to the hospital, she is accompanied by family and in a setting that provides a sense of security.

“*Everything is in Tsotsil, that’s why I like it better when a midwife [TBA] sees me” (User, 30 years, 4 children)*

*“At my house, I don’t spend anything [on transportation to the hospital] and there are less expenses. Besides, I have my whole family here, I have everything” (Non-user, 30 years, 4 children)*

From the point of view of users, the TBAs were available and accessible to providing them with care, in contrast to the health services.

*“The TBA is who is watching out for us 24 hours a day, and we trust her, it doesn’t matter what time it is, she always provides care for us” (User, 27 years, 3 children)*

TBAs are also capable of identifying the need for hospital care.

*“We went to call [the TBA] and she came quickly. When I started feeling badly she was the one to tell me it was better to go to the hospital so the doctors would evaluate me. Because I was going to miscarry the baby” (User, 30 years, 4 children)*

Users across widely expressed feeling more comfortable with the type of labor and delivery care they received from TBAs, due to the congruence with their traditional customs and practices.

*“We are accustomed to being given massages before labor; some TBAs warm our bellies with herbs so that we do not experience post-partum pain” (User, 30 years, 4 children)*

According to the non-users, the expected fee for the TBAs’ care provided in the home is accessible, as it is typically a voluntary amount based on the user’s ability to pay. This allows many women to seek out this type of care.

*“We actually do pay the TBA but not fixed amounts; we give them whatever we want to” (Non-user, 30 years, 4 children)*

Non-users expressed having greater trust in the TBAs because they themselves were women and had experienced childbirth.

*“We are used to the TBA, because there is more trust, because she is also a woman who has had children and knows very well how to tend to our needs” (Non-user, 24 years, 4 children)*

An advantage expressed by non-users was that the TBAs did not undress them and therefore they did not have to face the fear and shame of being nude while a stranger provided them with care.

*“I am sure she is going to take good care of me, and I am not ashamed with her, because she doesn’t take my clothes off like the doctors do at the hospital” (Non-user, 22 years, 3 children)*

#### Labor and delivery care at the Casa Materna (CM)

The users perceived that the availability of services at the CM did not resolve the economic barrier to accessing health services. For users to seek services at the CM, they had to incur the same expense in transportation as they would to seek care at the hospital.

*“We have to pay for the transportation and it’s not cheap, it’s very expensive” (User, 30 years, 4 children)*

Nonetheless, they recognize that the CM services are advantageous; they have timely access to the hospital and medical personnel in the event of a complication, without an additional cost for transportation.

*“The advantage of the CM is that when there is a complication it’s easier to be seen by a doctor, when we are already at the CM they can quickly see us there” (User, 30 years, 4 children)*

The users expressed that the TBAs recommended that pregnant women go to the CM for the birth, because if any complication were to present itself, the doctor could quickly evaluate them and in the event that they needed further care they would be admitted to the hospital.

*“I sought care at the CM because the TBA told me it’s better, it’s better for us to be seen at the CM because any type of complication and the doctor comes to see us or brings us quickly to the hospital” (User, 27 years, 3 children)*

The typical care practices used by TBAs at the CM before and after labor are valued and accepted by users, given that these practices coincide with the women’s own customs and habits.

*“Before labor, the TBA warms me with herbs so that I don’t suffer from too much pain. Normally she also warms us with some herbs after delivery so that we don’t feel pain” (User, 23 years, 4 children)*

Nonetheless, users mentioned structural aspects of the CM that could be improved. One of their observations was about the floor. On one hand they believed it was dangerous and could cause accidents; on the other hand they mentioned feeling embarrassed that their family members spoil the hygiene of the setting. Finally, users expressed that the CM building did not have the appropriate supports needed to hold onto during labor.

*“At the CM, starting with the floor it’s so slippery, and also there is nowhere for me to hold onto with the strength I need to push the baby, and I am also uneasy because it’s not my house” (User, 27 years, 3 children)*

The TBAs express that on some occasions doctors intervene when they are seeing women in the CM, interfering with their autonomy to provide services and ignoring user’s choice to be seen by a TBA. Similarly, the TBAs mention that the doctors do not wait for the evolution of labor and put pressure to move women to the hospital, despite knowing that, once in the hospital, TBAs are not allowed to provide care. Furthermore, the hospital reproduces practices that discourage the indigenous population, such as vaginal examination.

*“When we bring a women into the CM, when there is just a little time until the baby is born, the doctors take her and don’t allow her to give birth there [in the CM]…they don’t let us come in, only doctors can be with her. The women that they have taken always give birth in the hospital because when there is just a little bit of time until the baby is born, the doctors give a saline solution [for hydration] and then they take her to the area where they deliver the babies [at the hospital]” (TBA, 60 years, 30 years attending births)*

### Barriers and facilitators for labor and delivery at the Casa Materna (CM)

A summary of the primary barriers and facilitators of the available labor and delivery services at the CM based on the testimonies of users and non-users are summarized and presented in Table 2.

**Table 2:** Barriers and facilitators for labor and delivery at the *Casa Materna* for delivery services

*Categories*	*Availability*	*Accessibility*	*Acceptability/Quality*
Barriers	-Care is only available at the CM during the day	-The CM is isolated from local communities Transportation expenses to reach the CM -Food expenses while at the CM	-Must leave their other children at home to seek services at the CM -The CM facilities are not ideal for labor -Doctors interfere whit care given at the CM
Facilitators	-TBAs accompany women until labor is resolved at the CM	-Care at the CM is provided in the same language that women speak -Care at the CM provides benefits in the event of a complication because transportation to hospital facilities is rapid whit no additional cost	-At the CM procedure are conducted that women appreciate, such as massages before and after labor -During care provided at the CM the women are not undressed -During care provided at the CM invasive practices such as vaginal examination are not conducted -Services at the CM are provided by female personnel

## Discussion

This study permitted the exploration of the perceptions of indigenous women from the Chiapas highlands regarding the barriers and facilitators for the effectiveness of intercultural innovations in the internal context of health services where they were implemented. Labor and delivery services in the health sector institutions, particularly in the *Casa Materna* and Basic Community Hospital of San Andrés Larráinzar were compared to services provided by TBAs in their home communities. Important limitations of this study were the observation of a single hospital and CM, the reduced number of women interviewed as well as the need to rely on a trained translator. The findings should therefore be treated with some care, although results show a high degree of external validity.

The evidence presented suggests that from the perspective of indigenous women, the difficulty of communicating with health institution personnel is one of the primary barriers to service utilization, given that the majority of medical personal at health centers do not speak the same language as the indigenous users. Intercultural innovations have had limited impact in improving communication. According to Sachse et al. [14], health services should hire personnel who speak the local communities’ language to serve as translators for the women during care. However, having personnel who speak the local communities’ language is not sufficient. Based on findings from Nazar et al. [26] the participation of translators who do not share a bio-medical orientation does not guarantee the correct transmission of information given by the doctors to women, or in the case of requiring great precision of information, those individuals are unable to provide such precision. As such, not only should institutions have personnel that are able to communicate with users in their native language, it is also necessary to guarantee that the information given maintains the accuracy intended by the doctors.

In addition to language problems, indigenous women in Chiapas face economic limitations to accessing health services related to more than their transportation. The cost of food and transportation for family members that accompany the women to the hospital must also be considered. Intercultural innovations were not designed to help overcome the economic barriers. Several authors report findings coinciding with those found in our study, signaling that one of the primary reasons women do not seek care at institutional health services is due to lack of economic resources; they must consider not only their own expenses but also those of accompanying family members because these women do not seek health services alone [26, 27].

Another important element evidenced by the current study is that it appears that medical personnel are not always available at the health institutions, and as such the women fear seeking services and finding that personnel are absent. Intercultural innovations are likely to fail if their professional component is not consistently delivered and co-ordinated with the indigenous component. The literature documents that at the national level the absence of personnel in medical units is frequent, with higher intensity of absence on the night shift and during the weekend [26].

These factors may explain findings of others studies showing that health services utilization and coverage of interventions during pregnancy, delivery and postpartum are lowest among indigenous women in Mexico. For example, a study conducted with responses from 5766 women from the 2012 National Health and Nutrition Survey demonstrated gaps in the continuum of care during pregnancy and delivery in Mexico among indigenous women (0.759; CI95 %: 0.740–0.779) vs. 0.831 [0.823–0.838] in non indigenous women [28]. Additionally, previous studies had found that coverage of adequate antenatal care (timely, frequent and complete care) is lower among indigenous (59 %, CI:53;65) than non-indigenous (68 %, CI:66;70) women [29].

A finding that we should pay particular attention to is that indigenous women, specifically non-users of health services during delivery, only identified the need to seek health services in the event of a complication. Intercultural innovation failed to place emphasis on the need for prevention within a continuum of care. For health system performance, it is desirable for women to seek services at health institutions in a timely manner, even when there are no evident complications. Previous research, such as that conducted by Freyermuth et al. [30], show evidence of increased risk of fatal outcomes in labor and delivery services delivered outside of health institutions, given that warning signs during birth were not identified by women or their family members. These arguments provide the basis for the efforts on behalf of the health sector to increase awareness among the population that labor and delivery services should be provided by qualified personnel to minimize risk.

The lack of trust in health institutions is another one of the reasons reported by interviewees for not utilizing health services. Our findings suggest intercultural innovations have increased trust in professional services, although with limitations. These findings coincide with those reported by Sánchez Pérez, et al. [15], which found that lack of trust by women of health service providers was due to either the majority being men, or because the biomedical practices were not aligned with their customs. Such evidence was also illustrated by Valdez et al. [31], suggesting that routine medical practices could affect labor and delivery service utilization, and not only among indigenous women.

According to Valdez et al. [31], the maneuvers most frequently conducted during labor in institutional settings include vaginal examination, episiotomy, catheter insertion, enema and administering oxytocin infusions among others, which have been demonstrated to be unnecessary during labor. While our results did not address the need for interventions, it is clear that intercultural services should pay special attention to curbing unneeded interventions, which may harm trust even if the risk of harming patients is minimal The indigenous women interviewed mentioned their discomfort during different routine medical maneuvers that are practiced with them while receiving labor and delivery care at the health centers. As such, women experience cultural shock while receiving labor and delivery services due to the procedures and practices conducted without taking into account their beliefs, traditions and needs (for example, excessive use of instruments, IDU placement, undressing, using a language they are not fluent in, being alone, being seen by male personnel) [32].

Intercultural innovations failed to lift restriction on family members or TBAs being allowed into hospital facilities during labor. Women expressed the fear this hospital practice provokes. It has been documented that women suffer from anxiety, uncertainty and fear when seeking services at medical facilities due to facing a model of care that does not permit their family members of partners to remain close by, as opposed to care delivered at home where they can count on family accompaniment [33, 34]. Based on the testimonials collected during this study, women express feeling more secure, supported and calm with the presence of a family member while receiving services.

An important aspect to consider is the women’s identification of TBA services and their preference for being seen by a member of the same sex during labor, and moreover someone they can identify with culturally [26, 30]. Intercultural innovations failed to ensure that women doctors and nurses are charged with routine examinations. The basis for these preferences has been previously documented. The TBAs carry out important activities in regards to services for pregnant women, and many times their participation goes beyond labor and delivery care. They enact a symbolic role and provide not only physical care but social, spiritual care and trustworthy services based on the close familiar relationship that is perceived [33, 35].

The results of this study suggest the persistence of important limitations of institutional health services and intercultural innovations to attract indigenous women and encourage their utilization of services in an event as transformative as birth, from the context of the implementation of a policy of multiculturalism in the state. Women reported what would be violations of reproductive rights and the right to a safe motherhood. Evidence of this is the reported insertion of an IUD without previous consent, repeated vaginal examinations without informing the women about the need for such a procedure, limited access to information due to the unavailability of personnel who speak the local language [21, 31].

As it stands, biomedical practices and efforts to introduce intercultural innovations in health institutions may be reproducing a model of cultural incompetence that leads to the reduction, discrimination and exclusion of cultural minority groups, who additionally may be at a disadvantage [36, 37]. It is necessary to strengthen the capacity of the health system and review strategies that have been implemented with the aim of successfully incorporating practices with an intercultural focus for labor and delivery.

The Integrated Strategy to Accelerate the Reduction of Maternal Mortality in Mexico, which allows the incorporation of TBAs into health institutions, should be strengthened [2]. Based on this study’s findings, and considering the body of knowledge it is contributing to, TBAs associated with health services encounter medical hegemony and find themselves in subordinate positions with health professionals [38, 39]. Our results coincide with what other authors have described, that currently implemented policies in fact reproduce vertical models and frameworks, relationships of power-subordination among providers, etc. and are not reflective of models that fully integrate an intercultural vision [40]. Current policies only go so far as to promote the restructing of labor and delivery care for women, but without identifying pathways or processes for actually changing the practices that over-medicalize labor and delivery services in health institutions.

An example of how the Mexican health systems should respond to these findings may be the implementation and/or strengthening of the Project Casa de la Mujer Indígena or *Casas Model* [41] an initiative that brings together the local indigenous community, civil society organizations (NGO’s) and public institutions, in order to create a physical space to improve health care and patient satisfaction among indigenous women. This initiative intents to bridge the cultural distance between institutionalized, hegemonic forms of health care and indigenous forms of understanding and tending to health – especially reproductive health– within each community. Previous studies had identified some limitations and opportunity for improvement in this model. According to Pelcastre's evaluation [41], the model may be strengthened through the following elements: community ownership and participation; inter-agency partnership and networks; budget oversight and external advisor or advisory board, responsible for supporting training activities and generating applicable evaluation and research agendas [41].

## Conclusions

Evidence found in the study suggests the presence of important limitations for the utilization of institutionalized labor and delivery services within indigenous communities that purport to include intercultural adaptations. There is opportunity to improve on the depth and breadth of intercultural adaptations. Such models must sensitize personnel in charge of care to the needs, beliefs, practices and cultural preferences of indigenous women during labor. Greater emphasis should be given to the design of interventions that fully support human rights and gender equity and that continuously monitor and improve quality of care from an intercultural perspective.
